# Integrated Metagenomic and Lipidomic Profiling Reveals Dysregulation of Facial Skin Microbiome in Moderate Acne Vulgaris

**DOI:** 10.3390/microorganisms13122674

**Published:** 2025-11-24

**Authors:** Xiaoye Qi, Zhaoying Han, Jie Meng, Hongrui Zhao, Maoyuan Zhou, Meichao Wang, Shengze Kang, Qingying Shi, Hongyan Li, Fuping Lu, Huabing Zhao

**Affiliations:** Key Laboratory of Industrial Fermentation Microbiology, Ministry of Education, Tianjin Key Laboratory of Industrial Microbiology, College of Biotechnology, Tianjin University of Science and Technology, 9 TEDA 13th Street, Tianjin 300457, China; qixiaoye@sayyes.cloud (X.Q.); 19851820332@163.com (Z.H.); 15222522274@163.com (J.M.); zhaohr0330@163.com (H.Z.); 18716151928@163.com (M.Z.); 17732248404@163.com (M.W.); ksz000822@126.com (S.K.); shiqingying@tust.edu.cn (Q.S.); lihongyan@sayyes.cloud (H.L.); lfp@tust.edu.cn (F.L.)

**Keywords:** acne, metagenomics, lipidomics, skin microbiome

## Abstract

Acne vulgaris is a prevalent chronic inflammatory dermatosis primarily affecting the pilosebaceous units. Current therapeutic approaches often exhibit limited efficacy and high recurrence rates. To investigate the microbiome-related mechanisms of acne vulgaris, facial skin samples from 19 patients with moderate acne and 20 healthy individuals were analyzed using an integrated metagenomic and lipidomic profiling strategy. Metagenomic analysis revealed a significant reduction in microbial diversity (Chao index) in acne-affected skin compared to healthy controls (*p* < 0.001). The relative abundance of *Staphylococcus*, particularly *Staphylococcus epidermidis*, was significantly elevated in acne group (*p* < 0.05), while *Cutibacterium acnes* levels remained unchanged. Carbon metabolism pathways were enriched in the acne group (*p* < 0.05), predominantly driven by *Cutibacterium*, whereas other enriched metabolic pathways, such as ABC transporters and glycine, serine, and threonine metabolism (*p* < 0.05), showed a greater contribution from *Staphylococcus*. Virulence factors enriched in acne samples were primarily offensive in nature and largely attributed to *Staphylococcus*. Moreover, acne-associated microbiome exhibited a significantly higher prevalence of resistance genes against fluoroquinolones, fosfomycin, and triclosan (*p* < 0.05). Untargeted lipidomic analysis demonstrated significantly elevated total serum and triglyceride levels, along with a reduction in fatty acid chain length and a higher degree of saturation compared to the healthy group (*p* < 0.01). Specific triglycerides significantly enriched in the acne group, such as TG (15:0_14:0_16:0) + NH_4_, exhibited a significant positive correlation with *Staphylococcus*. This correlation is associated with elevated clinical erythema and melanin indices, suggesting that *Staphylococcus* is implicated in the development of acne-related inflammation. Additionally, *Thermus* exhibits negative correlations with acne-associated lipids and inflammatory parameters, potentially exerting a protective role. These findings suggest that *Cutibacterium* and *Staphylococcus* play differential yet synergistic roles in acne pathogenesis. The observed skin microbiome dysbiosis and lipid metabolic alterations provide novel insights into the pathophysiology of acne vulgaris, which may inform the development of targeted therapeutic strategies.

## 1. Introduction

Acne vulgaris is a prevalent, chronic inflammatory dermatosis of the pilosebaceous units, with lesions primarily occurring in sebaceous gland-rich areas, such as the face, accounting for approximately 99% of cases [1]. The condition imposes a substantial psychosocial burden, including diminished quality of life and self-esteem, as well as an increased risk of anxiety, depression, and suicidal ideation [2]. Its pathogenesis is multifactorial, involving altered sebum production, follicular hyperkeratinization, dysbiosis of the cutaneous microbiome, and complex inflammatory cascades [3].

Although microbial dysbiosis has been documented to be associated with acne development, the precise mechanistic connections between the functional roles of specific microbial taxa and disease pathology remain incompletely defined. *Cutibacterium acnes* contributes to acne pathogenesis via TLR2 recognition of bacterial ligands, activates pro-inflammatory signaling pathways involving the adapter protein myeloid differentiation primary response 88 and NF-κB, while adaptive immunity through Th1 and Th2 cells further amplifies inflammation [4,5]. However, evidence now indicates that the overall abundance of *C. acnes* may not differ in acne, yet specific phylotypes like IA1 are linked to inflammatory lesions [6,7,8]. Furthermore, some non-pathogenic subtypes appear protective, potentially by inhibiting microbial biofilm formation [9]. As a core commensal of human skin, *Staphylococcus epidermidis* normally maintains barrier function and promotes ceramide production [10]. Furthermore, certain *S. epidermidis* strains are increasingly recognized for their potential pathogenic roles, including biofilm formation and virulence factor expression [11].

While substantial research has revealed shifts within the acne microbiome, a predominant focus on classification and composition has provided only limited understanding at the functional level. The functional potential of the acne-associated microbiome, including the enrichment of specific metabolic pathways, the repertoire of virulence factors and their microbial origins, and the profile of antibiotic resistance genes, remains inadequately characterized. Moreover, the nature of microbial interactions, particularly those between *C. acnes* and *S. epidermidis* during acne development, is poorly understood [12].

Concurrently, alterations in skin surface lipids, which constitute a critical interface for host-microbe interactions, are recognized as key contributors to the disordered micro-environment in acne [13]. Lipidomic studies have identified alterations in the lipidome structure of patients with acne vulgaris, but how these metabolic changes interact with microbial functional pathways to drive disease remains unclear [14].

To address these limitations, we applied an integrated metagenomic and lipidomic approach to systematically profile the functional interplay between microbial pathways and lipid metabolism during acne development. This strategy moves beyond taxonomic associations to clarify how microbial processes mechanistically contribute to disease pathology. The resulting insights may reveal previously undefined mechanisms of acne pathogenesis and point to potential targets for microecological intervention.

## 2. Materials and Methods

### 2.1. Study Design

**Figure 1:**
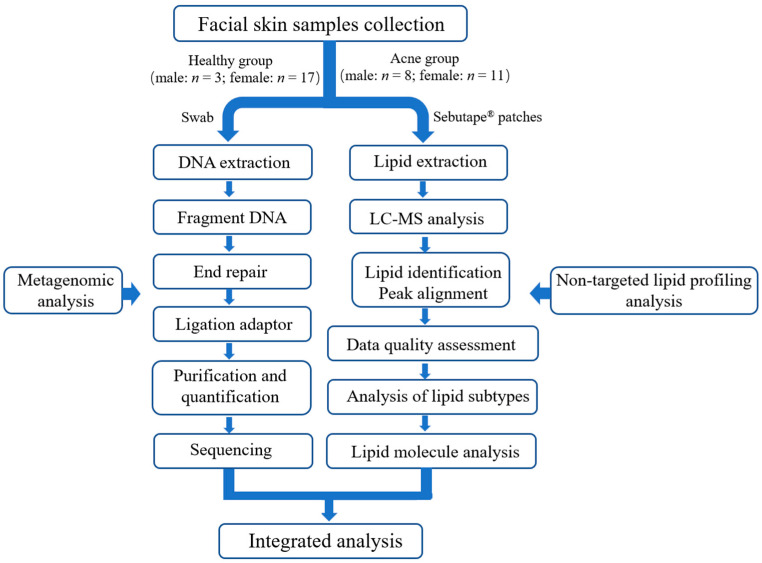
Chronological overview of the study, 20 healthy (male: *n* = 3; female: *n* = 17) and 19 acne participants (male: *n* = 8; female: *n* = 11) samples were collected, including swabs and Sebutape® patches. Swab samples were subjected to subsequent operations following the metagenomic analysis (left workflow in the figure). Sebutape® patche samples were processed subsequently according to the non-targeted lipid profiling analysis (right workflow in the figure). Finally, integrated analysis was performed on all acquired data.

The differences in facial microecology between individuals with acne and healthy controls were examined in this study. Thirty-nine participants aged 18 to 35 were enrolled, including 20 healthy volunteers and 19 patients with moderate acne. The calculation of the number of included participants was based on the formula: N = 2σ^2^ × f(α,β)/(μ1 − μ2)^2^, α = 0.01, β = 0.05 [15]. μ1 was set to 14.20, and μ2 was set to 10.37 [16]. The calculation showed that 18 participants were required per group at least. Participants were categorized into two groups: Healthy Skin (HS) and Acne Skin (AS). The HS group comprised 17 females and 3 males, with a mean age of 22.05 ± 2.46 years. The AS group comprised 11 females and 8 males, with a mean age of 22.00 ± 2.58 years. The age range of both groups is 19–26 years. All participants refrained from facial cleansing for 24 h before sample collection to standardize skin conditions. Facial microbiome and lipidome samples were then collected (Figure 1).

All procedures complied with the ethical standards approved by the Ethics Committee of Nankai University (approval number: NKUIRB2024216) and registered in the Chinese Clinical Trial Registry (Registration No.:ChiCTR2500101305; Registration Date: 23/04/2025). Written informed consent was obtained from all participants after explanation of study objectives and protocols. The study adhered to the Declaration of Helsinki and relevant national and international guidelines for human research [17]. 

### 2.2. Recruitment of Participants

A total of 39 participants aged 18 to 35 years were enrolled, including 19 patients with moderate acne (Pillsbury grade II–III) and 20 healthy volunteers. Inclusion criteria were as follows: moderate acne classified as Pillsbury grade II–III; lactic acid stinging test score below three; age over 18 years; no gender restrictions; informed consent obtained from participants and, where applicable, their families; willingness and ability to comply with study procedures and attend scheduled visits; completion of informed consent forms and provision of medical history; and avoidance of acne treatments affecting sample collection during the trial. Exclusion criteria were as follows: acne graded as Pillsbury I or IV; severe conglobate acne; other dermatological diseases such as drug-induced or occupational acne; lactic acid stinging test score ≥ 3; presence of atopic dermatitis, tinea, scarring, and skin fungal infections; use of acne treatments within two weeks prior to the study; long-term use of antibacterial cleansers; inability to comply with study requirements; and concurrent participation in other clinical trials.

### 2.3. Skin Sample Collection

Skin samples were obtained from acne lesions in patients and corresponding normal facial sites of healthy controls. A sterile cotton swab, moistened with normal saline, was wiped vertically and horizontally approximately 50 times over a ~6 cm^2^ area. The swab tip was then detached, placed in a sterile centrifuge tube, sealed, labeled, and stored at –80 °C for DNA extraction.

Skin surface metabolites were collected using Sebutape^®^ patches (Cuderm Corporation, Dallas, TX, USA) as described by Camera et al. [13] Each patch was applied for 5 min, removed, and stored at −80 °C for lipidomic analysis.

Biophysical parameters, including TEWL, sebum content, moisture content, erythema, and melanin, were measured using the Cutometer^®^ MPA 10 system (Courage+Khazaka electronic GmbH, Köln, Germany). Each parameter was assessed in triplicate per probe, and mean values were calculated for analysis.

### 2.4. Metagenomic Sequencing

DNA libraries were prepared using the QIAamp DNA Micro Kit (Qiagen, Hilden, Germany) and the NEXTFLEX Rapid DNA-Seq Kit (Bioo Scientific, Austin, TX, USA). Sequencing was performed on the Illumina NovaSeq platform (Illumina Inc., San Diego, CA, USA) at Majorbio Bio-Pharm Technology Co., Ltd. (Shanghai, China). Adapter sequences were trimmed from paired-end Illumina reads using the fastp software V1.0.1, and low-quality reads (length < 50 bp, quality score < 20) or those containing ambiguous bases (N) were discarded (https://github.com/OpenGene/fastp). Reads were aligned to the human genome using the Burrows-Wheeler Aligner, and any matches were removed. Contigs ≥ 300 base pairs (bp) were retained for subsequent gene prediction and annotation. Detailed statistical data analyses are provided in the Supplementary Materials and Methods.

### 2.5. Untargeted Lipidomic Analysis

Untargeted lipidomic analysis was performed by Applied Protein Technology Co., Ltd. (Shanghai, China) in three stages: (i) lipid extraction, (ii) liquid chromatography-tandem mass spectrometry (LC-MS/MS) analysis, and (iii) lipid identification [18]. Detailed statistical data analyses are provided in the Supplementary Materials and Methods.

### 2.6. Statistical Data Analysis

Experimental data are expressed as mean ± standard deviation (*n* = 3). For physical and chemical indicators, homogeneity of variance was first confirmed for normally distributed data. Differences between mean values were determined using one-way analysis of variance (ANOVA) followed by Tukey’s post hoc test in Origin 2018.

Bioinformatic analysis of the facial microbiota was performed on the Majorbio Cloud platform (https://cloud.majorbio.com) URL (accessed on 1 June 2025–1 October 2025). At the genus level, the Wilcoxon signed-rank test was used to evaluate differences in Shanno and Chao indices, with abundance calculated via the Reads Number method. The analysis of inter-group differences in alpha diversity was conducted using the Wilcoxon rank-sum test. Similarities among microbial communities were assessed by principal coordinate analysis (PCoA) based on Bray–Curtis dissimilarity using the Vegan v2.4.3 package. This was complemented by PERMANOVA non-parametric analysis to determine significant differences in microbial community structure between sample groups.

After alignment with various databases and annotation, the Wilcoxon rank-sum test (two-tailed, significance level 0.05) was applied. Multiple testing correction was performed using the false discovery rate method to identify differentially expressed species, functions, and genes.

Lipidomic data were analyzed using R and the online MetaboAnalyst platform (http://www.metaboanalyst.ca). Data were scaled to SD for multivariate analysis. Partial least squares-discriminant analysis (PLS-DA) was applied to assess lipidomic alterations among groups. The Wilcoxon rank-sum test and variable importance in projection (VIP) scores from pairwise PLS-DA were used to identify significantly altered metabolites, defined as VIP > 1 and *p* < 0.05 [19]. Volcano plots were generated using the ggplot2 R 3.6 package. A 200-permutation test was performed to assess overfitting risk in the PLS-DA model. Only confidently annotated metabolites were included in differential analysis. Spearman correlation heatmaps and correlation network analysis plots were used to evaluate the associations among differential lipids, clinical parameters, and species.

## 3. Results

### 3.1. Facial Skin Physicochemical Indexes

The study included 19 patients with acne and 20 healthy volunteers. Comparative analysis revealed no significant differences in facial moisture content between the Acne Skin (AS) group and the Healthy Skin (HS) group. However, the AS group exhibited significantly higher transepidermal water loss (TEWL), oil content, melanin index, and erythema index compared to the HS group (Table 1).

### 3.2. Abundance of Facial Microbiota Sequencing

Metagenomic sequencing was performed on facial swab samples from 19 patients with acne and 20 healthy volunteers. In acne lesions, the number of optimized reads after host sequence removal was significantly lower (*p* < 0.01) than in the corresponding sites of the HS group (Figure S1a). Following sequence assembly, gene prediction, and construction of non-redundant gene sets, the number of non-redundant microbial genes in the AS group was significantly lower (*p* < 0.01) than in the HS group (Figure S1b).

### 3.3. Analysis of Facial Microbiota Diversity

**Figure 2:**
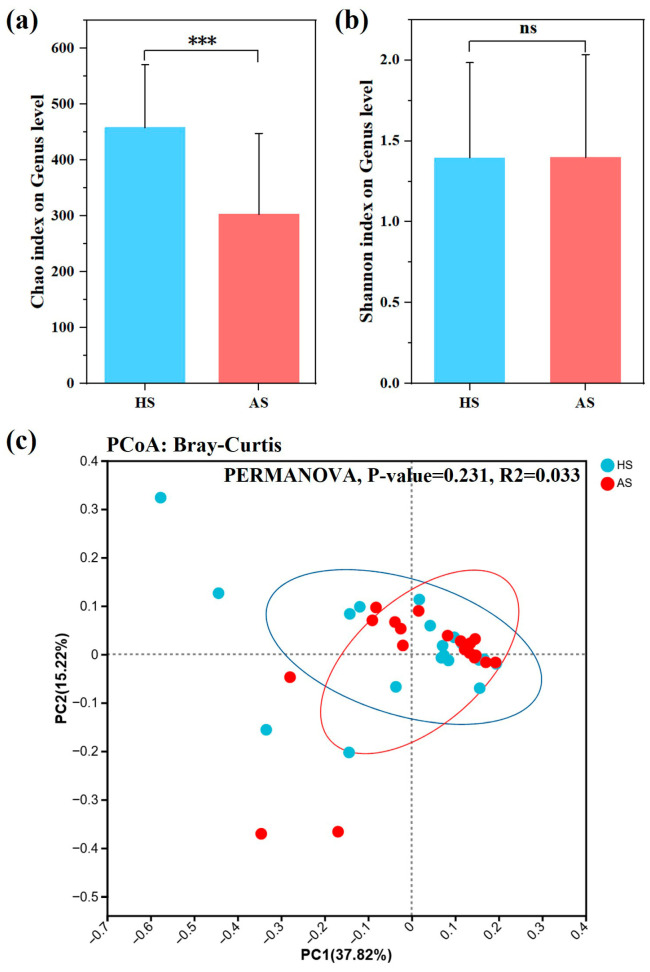
Microbial diversity in acne group (AS) and health group (HS). (**a**,**b**) Alpha diversity of bacterial taxa, analyzed using the Chao and Shannon indices, respectively. (**c**) Two-dimensional principal coordinate analysis (PCoA) plot depicting bacterial taxa diversity, calculated using the Bray–Curtis index. Asterisks indicate significance levels: *p* < 0.001 ‘***’ and ‘ns’ for non-significant.

Alpha (α) diversity was assessed using the Chao and Shannon indices. The Chao index, indicating bacterial richness, was significantly lower in the lesional skin of the AS group compared to the HS group (*p* < 0.001) (Figure 2a). The Shannon index, representing both richness and evenness, showed no statistically significant difference between groups (Figure 2b).

Microbial community clustering between groups was evaluated using Bray–Curtis indices (Figure 2c). PERMANOVA analysis showed no significant difference in β diversity between AS and HS groups (Bray–Curtis: *p* = 0.231; R^2^ = 0.033).

### 3.4. Taxonomic Composition of the Facial Microbiota in the Acne and Healthy Groups

**Figure 3:**
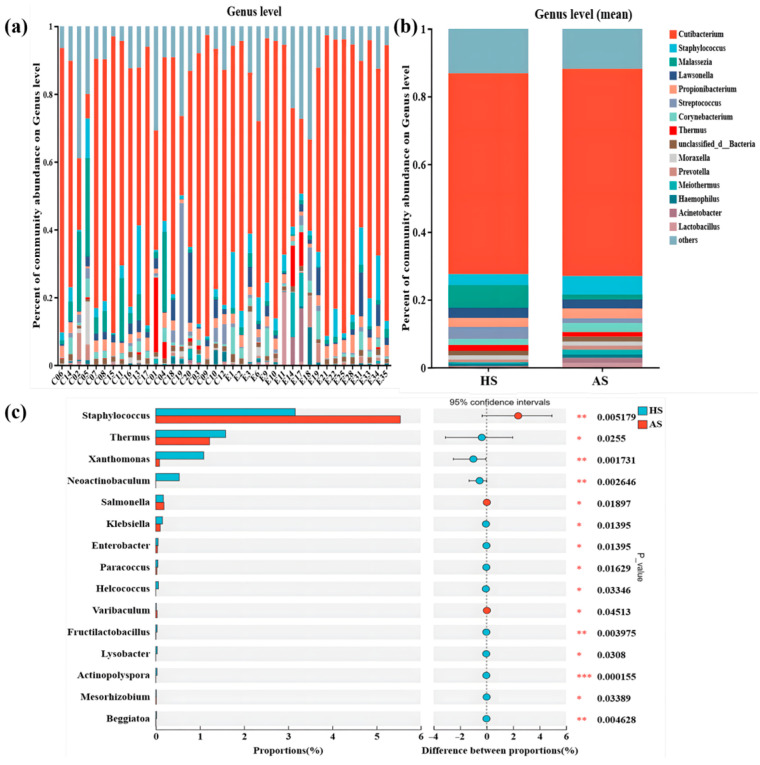
Composition of facial microorganisms in acne group (AS) and health group (HS). (**a**) Relative abundance of the main bacterial genera in the facial skin of each sample. (**b**) Relative abundance of the major bacterial genera in the acne and healthy groups. (**c**) Significant difference in relative abundance at the genus level between the two groups. Asterisks indicate significance level: *p* < 0.001 ‘***’; *p* < 0.01 ‘**’; *p* < 0.05 ‘*’.

Comprehensive analysis of the skin microbiota revealed 88 bacterial phyla, with Actinobacteriota (70.78%), Firmicutes (9.9%), and Proteobacteria (7.82%) being predominant. A total of 1555 microbial genera were identified; however, the microbial landscape was primarily shaped by a limited set of bacterial and fungal genera, several of which were typical skin commensals, including *Cutibacterium*, *Staphylococcus*, *Malassezia*, and *Streptococcus*. The ten most abundant microbial genera in acne samples were *Cutibacterium*, *Staphylococcus*, *Propionibacterium*, *Lawsonella*, *Corynebacterium*, *Acinetobacter*, *unclassified_d__Bacteria*, *Malassezia*, *Streptococcus*, and *Meiothermus* (Figure 3a,b). For healthy skin samples, the ten most abundant genera were *Cutibacterium*, *Malassezia*, *Streptococcus*, *Staphylococcus*, *Lawsonella*, *Propionibacterium*, *Thermus*, *Corynebacterium*, *unclassified_d__Bacteria,* and *Moraxella* (Figure 3a,b).

Furthermore, the relative abundances of *Thermus* (*p* = 0.026), *Xanthomonas* (*p* = 0.002), *Neoactinobaculum* (*p* = 0.039), *Klebsiella* (*p* = 0.014), *Enterobacter* (*p* = 0.014), *Paracoccus* (*p* = 0.016), *Helcococcus* (*p* = 0.033), *Fructilactobacillus* (*p* = 0.004), *Lysobacter* (*p* = 0.031), *Actinopolyspora* (*p* = 0.0002), *Mesorhizobium* (*p* = 0.034), and *Beggiatoa* (*p* = 0.005) were significantly lower in acne group. Conversely, *Staphylococcus* (*p* = 0.005), *Salmonella* (*p* = 0.019), and *Varibaculum* (*p* = 0.045) were significantly more abundant in acne group (Figure 3c). Notably, there was no significant difference in the relative abundances of *Cutibacterium* and *C. acnes* between acne and healthy groups (Figure 3c and Figure S2a). In contrast, compared with the healthy group, the relative abundances of *Staphylococcus* and *S. epidermidis* were higher in the acne group (Figure 3c and Figure S2b).

### 3.5. Functional Analysis of Facial Microbiota Genes in the Acne and Healthy Groups

A total of 192,705 non-redundant genes were mapped to 434 KEGG metabolic pathways. KEGG annotation profiles for each group were obtained by comparison with the KEGG database. At pathway level 1, the majority of genes were associated with metabolism, followed by genetic information processing, environmental information processing, cell processing, and human disease (Figure S3a). At level 2, global and overview maps accounted for the largest proportion, followed by carbohydrate, amino acid, cofactor and vitamin metabolism, and membrane transport (Figure S3b). At level 3, metabolic pathways predominated, followed by secondary metabolite biosynthesis, microbial metabolism in diverse environments, cofactor biosynthesis, amino acid biosynthesis, and carbon metabolism (Figure S3c). Overall, the primary functions of the facial microbiota were related to metabolism, genetic information processing, and environmental information processing.

**Figure 4:**
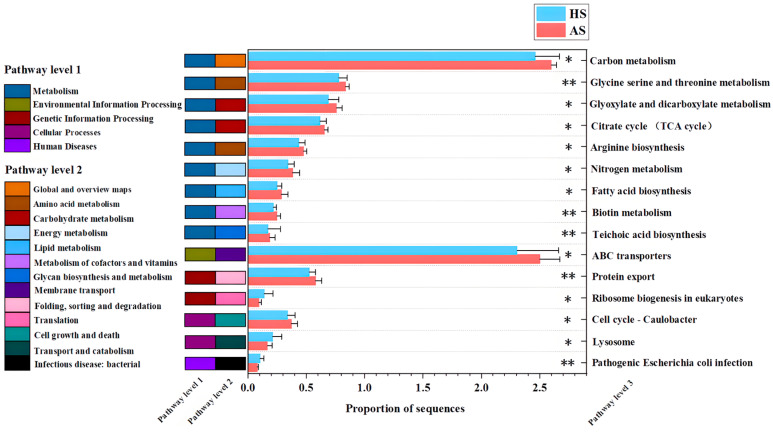
Differential KEGG pathway of level 3 between acne group (AS) and healthy group (HS). Asterisks indicate significance level: *p* < 0.01 ‘**’; *p* < 0.05 ‘*’.

Analysis of differential metabolic pathways revealed significantly higher relative abundances of carbon metabolism, glycine-serine-threonine metabolism, glyoxylate and dicarboxylate metabolism, the citrate cycle (TCA cycle), arginine biosynthesis, nitrogen metabolism, fatty acid biosynthesis, biotin metabolism, teichoic acid biosynthesis, ABC transporters, protein export, and Caulobacter cell cycle in the acne group compared with the healthy group (*p* < 0.05). In contrast, lysosome, ribosome biogenesis in eukaryotes, and pathogenic *Escherichia coli* infection pathways were significantly lower in the acne group (*p* < 0.05) (Figure 4).

Contributions of different bacterial genera to the differential KEGG level 3 pathways identified between the Acne Skin (AS) and Healthy Skin (HS) groups were further evaluated (Figure S4). Genus-level contributions to enriched KEGG level 3 pathways in the AS group showed that *Cutibacterium* contributions in carbon metabolism increased from 3% in the HS group to 35% in the AS group. This suggests an enhanced role for *Cutibacterium* in carbon metabolism during acne pathogenesis, potentially mediated through lipase-driven hydrolysis of triglycerides into glycerol and free fatty acids [12]. In contrast, *Staphylococcus* exhibited greater contributions to other differentially enriched pathways in the AS group, particularly ABC transporters and glycine-serine-threonine metabolism. These pathways are implicated in nutrient uptake, stress response, and the production or secretion of virulence-associated molecules [20]. These findings suggest a potential functional synergy between *Cutibacterium* and *Staphylococcus* in acne development, with *Cutibacterium* predominantly enhancing carbon metabolism and *Staphylococcus* contributing to complementary metabolic and transport processes.

Further exploration of functional properties involved assigning significantly detected genes to the Cluster of Orthologous Groups (COG) database. The major COG categories included “Metabolism” ([G] carbohydrate transport and metabolism, [E] amino acid transport and metabolism, [H] coenzyme transport and metabolism, [C] energy production and conversion), “Cellular processes and signaling” ([M] cell wall/membrane/envelope biogenesis), and “Information storage and processing” ([K] transcription) (Figure S5a). In total, 461 COGs differed between groups. In the acne group, the relative abundances of membrane protein and peptidoglycan biosynthesis (COG0427, COG1968), transmutation gene (COG3328), DNA repair (COG1061), and pyrimidine biosynthesis (COG0540) were elevated. Conversely, the acne group showed significantly lower abundances of ribosomal proteins L1 (COG0081, COG0090), unsaturated fatty acid degradation (COG1024), serine protease (COG1404), and ATPase activity (COG0484) compared with the healthy group (Figure S5b).

### 3.6. Analysis of Virulence Factors and Drug Resistance Genes in the Facial Microbiota of the Acne and Healthy Groups

**Figure 5:**
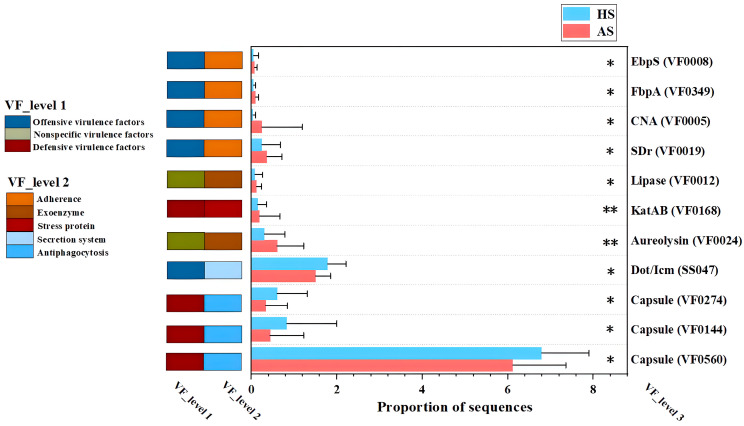
Differential virulence factor between acne group (AS) and healthy group (HS). Asterisks indicate significance level: *p* < 0.01 ‘**’; *p* < 0.05 ‘*’.

Figure 5 illustrates the differential virulence factors between the acne group (AS) and the healthy group (HS). Among the virulence factor (VF) genes enriched in the acne group, aggressive virulence genes predominated, whereas defensive virulence genes were more prevalent among those enriched in the healthy group. The enrichment of adhesion factors, such as EbpS (Elastin-binding protein, VF0008), FbpA (Fibronectin-binding protein, VF0349), CAN (Collagen-Binding Protein, VF0005), and SDr (Serine-Aspartate Repeat proteins, VF0019) in the acne group suggests an enhanced capacity for bacterial attachment to host extracellular matrix components, a critical first step in colonization and biofilm formation [21,22,23,24]. The enrichment of the extracellular enzymes Lipase (VF0012) and Aureolysin (VF0024) points to mechanisms of tissue invasion and immune evasion. Lipase may facilitate bacterial survival and persistence within the lipid-rich sebaceous environment by hydrolyzing sebum triglycerides, simultaneously releasing pro-inflammatory free fatty acids [25]. Aureolysin, a metalloprotease, can cleave host antimicrobial peptides and surface proteins, disrupting the epidermal barrier and potentially enabling deeper bacterial invasion [26]. Furthermore, the catalase KatAB (VF0168) promotes intracellular survival by neutralizing reactive oxygen species produced by host phagocytes, underscoring its role in sustaining bacterial persistence during host immune responses [27,28]. In contrast, the VFs enriched in the healthy group were predominantly defensive. Three capsule-encoding genes (VF0274, VF0144, and VF0560) are associated with antiphagocytic activity, facilitating evasion of the host immune system by protecting bacteria from opsonophagocytosis and serum-mediated killing [29]. Additionally, the Dot/Icm secretion system (Type IV Secretion System, SS047) plays a critical role in inducing apoptosis in human macrophages, representing a strategy to subvert host immune clearance [30].

To further identify which strains are responsible for producing the acne group-enriched virulence factors, a mapping of the virulence factors to the strains was conducted (Figure S6). The mapping analysis revealed that the virulence factors (EbpS, FbpA, CAN, SDr, Lipase, KatAB, and Aureolysin) enriched in the facial metagenomes of the acne group were predominantly derived from *Staphylococcus* rather than *Cutibacterium*. These findings suggest that *Staphylococcus* may be directly linked to acne-related immune responses.

In addition, the acne group and the healthy group had a total of 21 antibiotic classes. A bar chart was then generated to illustrate the difference in the relative abundance of these antibiotic classes between the two groups. The results showed that the resistance genes of fluoroquinolne antibiotic class, fosfomycin and triclosan in the acne group were significantly higher than those in the healthy group (*p* < 0.05) (Figure S7).

### 3.7. Differences in Facial Skin Lipids Between the Acne and Healthy Groups

Principal component analysis (PCA) and partial least squares discriminant analysis (PLS-DA) were used to distinguish lipid classes between AS and HS groups. PCA showed separation between groups (R^2^X = 0.513, Figure S8a), and PLS-DA confirmed a distinct separation (R^2^X = 0.387, R^2^Y = 0.685, Q^2^ = 0.507, Figure S8b).

**Figure 6:**
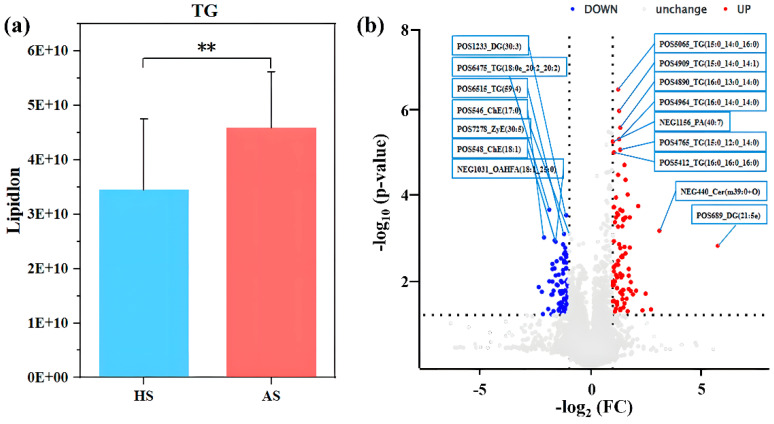
Analysis of lipid difference between acne group (AS) and healthy group (HS) (**a**) Relative abundance histogram of facial triglycerides. (**b**) Volcano plot of lipid changes between the two groups. Asterisks indicate the significance level: *p* < 0.01 ‘**’.

Bar chart analysis revealed significantly higher triglyceride levels in the AS group than in the HS group (*p* < 0.05) (Figure 6a), with no significant differences in other lipid subgroups. Volcanic plot analysis identified dominant differential lipid species, showing that triglycerides in the AS group had shorter chain lengths and a higher degree of saturation compared to those in the HS group (Figure 6b). The MS1 and MS2 spectra of the top 10 differential lipids between the two groups are presented in Figure S9.

### 3.8. Relationship Between Facial Skin Microbiome, Differential Skin Lipids, Clinical Factors in Acne and Healthy Groups

**Figure 7:**
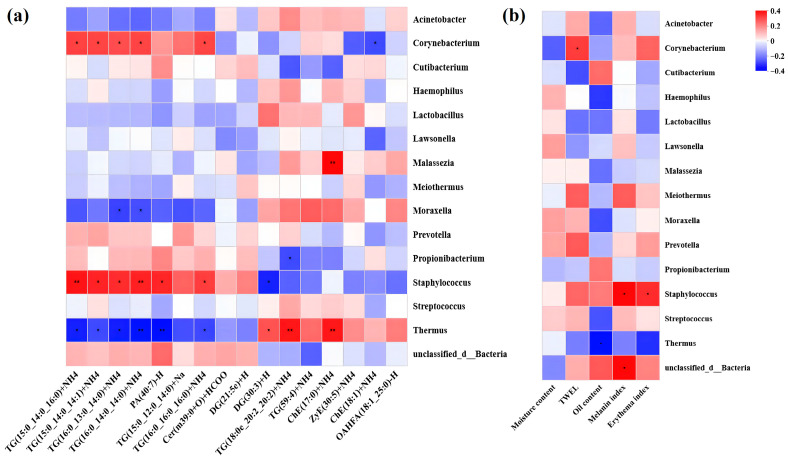
Associations between differential skin lipids, clinical parameters, and microbial taxa in the skin of acne and healthy groups. (**a**) Heatmaps generated by Spearman correlation analysis illustrating the relationship between differential skin lipids and skin microbes. (**b**) Heatmaps from Spearman correlation analysis depicting correlations between various clinical parameters and microbiome composition. Asterisks indicate the significance level: *p* < 0.01 ‘**’; *p* < 0.05 ‘*’.

Correlation analysis examined relationships among skin microbes, differentially abundant lipids, and physicochemical indices, visualized in a heat map. *Corynebacterium* and *Staphylococcus* showed a positive correlation with the lipids enriched in the AS group. As illustrated in the heatmap (Figure 7a), distinct association patterns were observed between specific bacterial genera and lipid species. *Staphylococcus* exhibited positive correlations with multiple triglycerides (TGs) that were elevated in the AS group, including TG (15:0_14:0_16:0) + NH_4_, TG (15:0_14:0_14:1) + NH_4_, TG (16:0_13:0_14:0) + NH_4_, TG (16:0_14:0_14:0) + NH_4_, and TG (16:0_16:0_16:0) + NH_4_. *Staphylococcus* was additionally positive correlated with phosphatidic acid PA (40:7) + Na. In contrast, *Thermus* showed consistent negative correlations with this set of TGs and PA (40:7) + Na, while Moraxella was negatively correlated specifically with TG (16:0_13:0_14:0) + NH_4_ and TG (16:0_14:0_14:0) + NH_4_.

Regarding clinical parameters (Figure 7b), *Corynebacterium* abundance was positively correlated with TEWL. *Staphylococcus* showed positive associations with both the melanin and erythema indices, whereas *Thermus* was negatively correlated with skin oil content.

**Figure 8:**
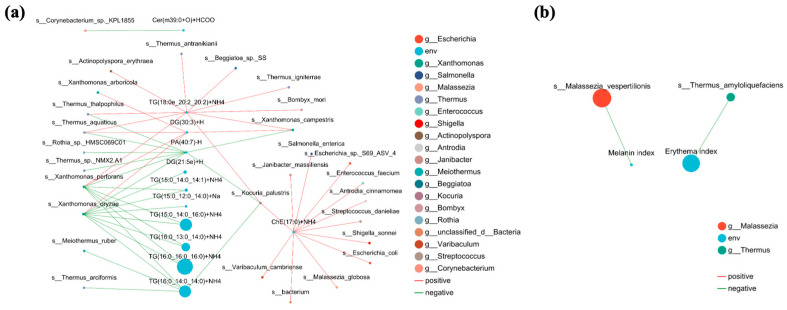
Correlation network analysis of skin lipids, clinical parameters, and microbial taxa in the skin of acne and healthy groups. (**a**) Network analysis between differential skin lipids and skin microbes. (**b**) Network analysis between various clinical parameters and microbiome composition.

Furthermore, two correlation networks (Figure 8) were constructed to reveal the multidimensional interactions among differential skin lipids, microbial taxa, and clinical parameters. Consistent with the elevated triglyceride levels in the acne skin (AS) group (Figure 6a, *p* < 0.05) and their characteristics of short chain lengths and high saturation (Figure 6b), the major AS enriched TGs, including TG (16:0_16:0_16:0) + NH_4_, TG (16:0_14:0_14:0) + NH_4_, TG (15:0_14:0_16:0) + NH_4_, TG (16:0_13:0_14:0) + NH_4_, TG (16:0_12:0_14:0) + NH_4_, and TG (15:0_14:0_14:1) + NH_4_, exhibited negative correlations with multiple bacterial taxa, particularly *Xanthomonas*. Lipid molecules enriched in the healthy skin (HS) group, such as phosphatidic acid PA (40:7) + Na, DG (30:3) + H, and TG (180e_20:2_20:2) + NH4, showed positive correlations with species represented by the *Thermus*. Figure 8b focuses on the associations between microbial taxa and clinical physicochemical indices. *Thermus_amyloliquefaciens* showed a negative correlation with the erythema index, and *Malassezia_vespertilionis* exhibited a negative correlation with the melanin index. This complements the negative correlation between *Thermus* and skin oil content observed in Figure 7b, indicating that *Thermus* may act as a “protective” microbe that could potentially alleviate lipid accumulation and the clinical symptoms of acne.

## 4. Discussion

By integrating metagenomic sequencing with lipidomic profiling, this study elucidated how alterations in microbial function and lipid metabolites in acne-affected skin contribute to disease pathogenesis. The findings thereby shift the focus from taxonomic description to a functional understanding of microecological dysbiosis in acne.

Metagenomic sequencing revealed a substantial reduction in the optimized reads and unique sequences in the acne group post-de-hosting, compared with the healthy cohort. This decline indicates a notable decrease in facial microbial content (Figure S1), analogous to findings in psoriasis [31]. Inflammatory processes in acne lesions contribute to host cell damage and rupture, leading to the release of substantial host DNA. This elevates the human DNA back-ground and obscures microbial community signals [32]. Additionally, antimicrobial molecules present in acne lesions, such as hBD-2, S100A7, human neutrophil peptides (HNP) 1-3, and granulin, further enhance the antimicrobial milieu [33]. Concurrently, the acne group displayed significantly elevated skin lipid levels (Table 1). Abnormal sebum secretion disrupts microbial community equilibrium, and changes in the microenvironment similarly influence microbial abundance [34]. These factors together explain the observed reduction in microbial content.

Regarding ɑ-diversity, the acne group showed significantly reduced microbial richness (Chao index), consistent with other inflammatory conditions such as psoriasis [31], atopic dermatitis [35], and Crohn’s disease [36] (Figure 2a). This decrease in species richness is accompanied by a reduction in the total number and variety of microorganisms. The Chao index’s sensitivity to rare taxa suggests that low-abundance species are disproportionately affected in lesions. The unchanged Shannon index indicates that overall community diversity and evenness remain comparable to healthy skin (Figure 2b). These findings imply that remaining taxa might sustain ecological roles and interrelations via adaptive mechanisms, including enhanced metabolism, membrane protein and peptidoglycan biosynthesis, etc. In the analysis of microbiome β-diversity, variability among distinct groups was examined using principal component analysis (Figure 2c). The findings of this study indicate that facial microecology constitutes a relatively stable environment not significantly influenced by moderate acne. In contrast, patients with severe acne exhibit substantial deviations in microbial community structure compared to the normal state [37]. This indicates that facial microecology may maintain relative equilibrium through dynamic regulation during mild to moderate inflammation, whereas severe acne triggers significant dysbiosis.

Taxonomic composition analysis revealed that the dominant genera remained consistent between the healthy group and the acne group, with no significant changes in species composition (Figure 3a,b), which is consistent with previous findings [38]. The stable predominance of species indicates preserved microbial community structure in moderate acne. In the analysis of differential microorganisms, the relative abundance of *Cutibacterium* and *C. acnes* remained unchanged between groups (Figure S2). Previous studies demonstrate *C. acnes* proliferation is not the primary cause of acne, as patients with acne do not harbor more *C. acnes* in follicles than unaffected individuals [3]. Notably, the relative abundances of *Staphylococcus* and *S. epidermidis* were significantly increased in the acne group (Figure 3c and Figure S2b), a finding consistent with prior literature [10,39]. The abundance of increased *Staphylococcus* and *S. epidermidis* in mild-to-moderate acne may relate to elevated biofilm formation, providing a protective niche [11,40].

Facial skin from the acne group exhibited a significant increase in the relative abundance of KEGG-defined metabolic pathways compared to healthy skin (Figure 5). These alterations disrupt skin homeostasis and enhance immune responses. Elevated pathways, including carbon metabolism and ABC transporter proteins, supply carbon and energy, enabling microorganisms to maintain an ‘active state’ locally. Notably, *C. acnes* produces short-chain fatty acids (SCFAs) during carbon metabolism, which under inflammatory conditions activate the NLRP3 inflammasome, leading to the release of IL-1β and a pro-inflammatory cytokine response [41]. Additionally, enrichment of carbon metabolic pathways promotes lipid synthesis and sebum secretion [42], increasing the risk of follicular sebaceous gland duct occlusion, thereby contributing to acne pathogenesis. Supporting this, the acne and insulin resistance study reported enrichment of the ABC transporter protein pathway in patients with acne, in line with the study results [43]. Bacterial ABC transporters facilitate antibiotic efflux and secretion of toxic compounds, contributing to bacterial resistance. The results demonstrate that *Staphylococcus* contributed more to the ABC transporter protein pathway and differential virulence factors, suggesting its potential involvement in acne development and antibiotic resistance (Figure S4).

In the GO database analysis, the acne group exhibited a significantly higher relative abundance of proteins associated with peptidoglycan synthesis, DNA repair, and pyrimidine biosynthesis compared to the healthy group (Figure S5b). The positive correlation between the increased abundance of *Staphylococcus* and the availability of substrates for bacterial growth and replication further supports the strong association between *Staphylococcus* and acne pathogenesis. Therefore, targeting the suppression of *Staphylococcus* proliferation may hold considerable therapeutic potential for acne management.

Virulence factor profiling revealed that the acne group was enriched in aggressive virulence genes, whereas defensive virulence genes predominated in the healthy group (Figure 5). Under homeostatic conditions, microbial communities maintain equilibrium with the host, with defensive virulence factors contributing to immune tolerance by preventing excessive immune activation and facilitating microbial colonization and evasion of host clearance [44]. For instance, certain *C. acnes* strains express surface proteins or polysaccharides that mask antigenic determinants, enabling immune evasion and symbiotic coexistence with the host [45,46]. However, during acne pathogenesis, virulence factors contribute to disease progression by mechanisms including the destruction of host cells, induction of inflammatory responses, and modulation of host immunity via adhesion, colonization, and biofilm formation [40]. Notably, the virulence factors enriched in the acne group were predominantly derived from *Staphylococcus*, indicating an association between this genus and acne pathogenesis (Figure S6). Comprehensive genomic analyses of *C. acnes* and *S. epidermidis* from public databases reveal that *C. acnes* lacks most virulence genes present in *S. epidermidis* [12]. This finding, together with our results (Figure S6), collectively supports the view that *S. epidermidis* appears to play a more significant role than *C. acnes* in inducing inflammation-related markers [47].

Lipidomic analysis revealed significantly elevated levels of total lipids and triglycerides (TGs) in the facial skin of acne patients, accompanied by shorter fatty acid chain lengths and a higher degree of saturation (Figure 6). This finding aligns with previous studies which have reported a significant increase in triglyceride content and a higher degree of saturation in the skin surface lipids of individuals with acne [14,48]. Furthermore, the accumulation of TGs may provide growth substrates for certain microorganisms, thereby influencing microbial community structure and metabolic activity [12]. Correlation analysis further demonstrated that specific triglycerides significantly enriched in the acne group, such as TG (15:0_14:0_16:0) + NH_4_, exhibited a significant positive correlation with *Staphylococcus*, while showing negative correlations with commensal genera like Thermus (Figure 7a). Regarding clinical parameters, *Staphylococcus* abundance was positively correlated with both the melanin and erythema indices (Figure 7b), suggesting its potential involvement in acne-associated inflammatory responses and pigmentation. This finding supports the ongoing re-evaluation of the role of *Staphylococcus* in acne pathogenesis, indicating that it is not merely a commensal but may adopt a pro-inflammatory role under specific conditions [11]. Collectively, these results indicate that acne-associated skin is characterized by a co-occurring increase in *Staphylococcus* along with a distinct set of triglycerides, whereas commensal genera such as Thermus are associated with a healthier lipid profile and skin homeostatic condition.

The nature of the interaction between *C. acnes* and *S. epidermidis* in acne pathogenesis, whether antagonistic or cooperative, the findings across different studies remain inconsistent [49,50]. We hypothesize that *S. epidermidis* may not be confined to the previously acknowledged beneficial effects, such as antagonizing *C. acnes* [51], but may also exert a pro-inflammatory effect [52] and synergize with *C. acnes* to contribute to the pathogenesis of acne vulgaris. Specifically, our metagenomic analysis revealed no significant difference in the relative abundance of *C. acnes* between acne group and healthy group, whereas *S. epidermidis* was significantly enriched in acne group (Figure S2). More importantly, the virulence factors were contributed by *Staphylococcus*, not *Cutibacterium*. These findings suggest a re-evaluation of the historical emphasis on *C. acnes* as the primary instigator of inflammatory damage and implicate *S. epidermidis* is a player in the immune responses associated with acne. Furthermore, metagenomic results revealed significant enrichment of carbon metabolic pathways in the acne group compared with the healthy group, with *Cutibacterium* contributing more to carbon metabolism and *Staphylococcus* contributing more to other enriched metabolic pathways.

Despite the insights provided by this integrated multi-omics approach, several limitations of the present study must be acknowledged. First, the limited cohort size (*n* = 39) and narrow age range (19–26 years) may constrain the generalizability of our findings. Second, the cohort had an imbalanced sex distribution (HS: 17 females and 3 males; AS: 11 females and 8 males), which prevented a robust analysis of sex-related factors. Third, host intrinsic factors such as body mass index and systemic metabolic status were not assessed. Furthermore, while the functional inferences drawn from metagenomic data are valuable, they remain largely predictive.

## 5. Conclusions

By integrating metagenomic and lipidomic profiling, this study extends beyond traditional microbial taxonomy to provide a functional and ecological perspective on the acne microbiome. Our data suggest that acne is characterized by a co-occurring dysbiosis of the microbiome and lipidome. In this process, *Cutibacterium* and *Staphylococcus* may collaboratively contribute to pathogenesis: *Cutibacterium* potentially drives changes in skin lipids, while *Staphylococcus* is associated with virulence and pro-inflammatory processes. These findings are crucial not only to better understand the pathophysiology in moderate acne, but also to inform the development of targeted therapies, such as selective anti-staphylococcal agents [32], precision probiotics [53]. By focusing on the balance of the skin microbiota, ecological strategies stand out as a promising therapeutic approach for early-stage acne, while offering the potential to reduce the side effects and drug resistance associated with conventional therapies like retinoids and antibiotics.

## Figures and Tables

**Table 1:** Facial skin physicochemical indexes in acne (AS) and healthy (HS) groups.

Physicochemical Indexes	HS (Mean ± SD)	AS (Mean ± SD)	*p*-Value
Moisture content	79.49 ± 14.34	74.62 ± 18.11	0.24
TWEL	16.80 ± 3.77	25.81 ± 7.20	<0.001
Oil content	17.30 ± 11.95	35.27 ± 16.67	<0.001
Melanin index	20.71 ± 22.20	57.20 ± 34.57	<0.001
Erythema index	227.34 ± 51.77	353.84 ± 67.50	<0.001

## Data Availability

The original contributions presented in this study are included in the article/Supplementary Material. Further inquiries can be directed to the corresponding author.

## Supplementary Materials

The following supporting information can be downloaded at: https://www.mdpi.com/article/10.3390/microorganisms13122674/s1, Figure S1: Comparison of sequencing abundance between acne group (AS) and healthy group (HS); Figure S2: Relative abundances of *Cutibacterium acnes* and *Staphylococcus epidermidis* in AS and HS groups; Figure S3: KEGG metabolic pathway composition in acne group (AS) and healthy group (HS); Figure S4: Contribution of bacterial genera to differences in KEGG pathways at level 3 between the acne group (AS) and healthy group (HS); Figure S5: COG composition of facial microorganisms in acne group (AS) and health group (HS); Figure S6: Contribution of bacterial genera to differences in virulence factors between the acne group (AS) and healthy group (HS); Figure S7: Differential antibiotic class composition between acne group (AS) and healthy group (HS); Figure S8: Metabolic profile differentiation between acne group (AS) and healthy group (HS); Figure S9: MS1 and MS2 Spectra of the Top 10 differential lipids between acne group (AS) and healthy group (HS). Refs [54,55,56,57,58,59,60,61] are included in Supplementary Materials.

## References

[1] Chen H., Zhang T.C., Yin X.L., Man J.Y., Yang X.R., Lu M. (2022). Magnitude and temporal trend of acne vulgaris burden in 204 countries and territories from 1990 to 2019: An analysis from the Global Burden of Disease Study 2019. Br. J. Dermatol..

[2] Lai Y., Fan M., Fan X., Chen J., Xiang L.F., Ma Y. (2025). Progress on Multiomics Research on Acne Vulgaris: A Literature Review. J. Investig. Dermatol..

[3] Dréno B., Pécastaings S., Corvec S., Veraldi S., Khammari A., Roques C. (2018). *Cutibacterium acnes* (*Propionibacterium acnes*) and acne vulgaris: A brief look at the latest updates. J. Eur. Acad. Dermatol. Venereol..

[4] Kawai T., Akira S. (2007). TLR signaling. Semin. Immunol..

[5] Sanford J.A., O’Neill A.M., Zouboulis C.C., Gallo R.L. (2019). Short-Chain Fatty Acids from *Cutibacterium acnes* Activate Both a Canonical and Epigenetic Inflammatory Response in Human Sebocytes. J. Immunol..

[6] Mias C., Mengeaud V., Bessou-Touya S., Duplan H. (2023). Recent advances in understanding inflammatory acne: Deciphering the relationship between *Cutibacterium acnes* and Th17 inflammatory pathway. J. Eur. Acad. Dermatol. Venereol..

[7] Paugam C., Corvec S., Saint-Jean M., Le Moigne M., Khammari A., Boisrobert A., Nguyen J., Gaultier A., Dréno B. (2017). *Propionibacterium acnes* phylotypes and acne severity: An observational prospective study. J. Eur. Acad. Dermatol. Venereol..

[8] Borrel V., Gannesen A.V., Barreau M., Gaviard C., Duclairoir-Poc C., Hardouin J., Konto-Ghiorghi Y., Lefeuvre L., Feuilloley M.G.J. (2019). Adaptation of acneic and non acneic strains of *Cutibacterium acnes* to sebum-like environment. Microbiologyopen.

[9] Nakamura K., O’Neill A.M., Williams M.R., Cau L., Nakatsuji T., Horswill A.R., Gallo R.L. (2020). Short chain fatty acids produced by *Cutibacterium acnes* inhibit biofilm formation by *Staphylococcus epidermidis*. Sci. Rep..

[10] Dreno B., Dekio I., Baldwin H., Demessant A.L., Dagnelie M.A., Khammari A., Corvec S. (2024). Acne microbiome: From phyla to phylotypes. J. Eur. Acad. Dermatol. Venereol..

[11] Fournière M., Latire T., Souak D., Feuilloley M.G.J., Bedoux G. (2020). *Staphylococcus epidermidis* and *Cutibacterium acnes*: Two major sentinels of skin microbiota and the influence of cosmetics. Microorganisms.

[12] Yu T., Xu X., Liu Y., Wang X., Wu S., Qiu Z., Liu X., Pan X., Gu C., Wang S. (2024). Multi-omics signatures reveal genomic and functional heterogeneity of *Cutibacterium acnes* in normal and diseased skin. Cell Host Microbe.

[13] Camera E., Ludovici M., Tortorella S., Sinagra J.L., Capitanio B., Goracci L., Picardo M. (2016). Use of lipidomics to investigate sebum dysfunction in juvenile acne. J. Lipid Res..

[14] Cheng Y., Sun Q., Gao J., Liu Q., Tian H., Ding H., Qiao J., Chen H. (2025). Quantitative lipidomics profiling of skin surface lipids and skin barrier function evaluation in patients with acne vulgaris. Arch. Dermatol. Res..

[15] Pocock S.J. (2013). Clinical Trials: A Practical Approach.

[16] Xiao Y. (2022). High—Throughput Sequencing Analysis of the Facial Microbiota Before and After 2% Supramolecular Salicylic Acid Treatment in Patients with Moderate Acne Vulgaris. Master’s Thesis.

[17] Mayer W., Weibel M., De Luca C., Ibragimova G., Trakhtman I., Kharaeva Z., Chandler D.L., Korkina L. (2023). Biomolecules of Fermented Tropical Fruits and Fermenting Microbes as Regulators of Human Hair Loss, Hair Quality, and Scalp Microbiota. Biomolecules.

[18] Yuan Y., Xu F., Jin M., Wang X., Hu X., Zhao M., Cheng X., Luo J., Jiao L., Betancor M.B. (2021). Untargeted lipidomics reveals metabolic responses to different dietary n-3 PUFA in juvenile swimming crab (*Portunus trituberculatus*). Food Chem..

[19] Wei J., Dai W., Pan X., Zhong Y., Xu N., Ye P., Wang J., Li J., Yang F., Luo J. (2023). Identifying the Novel Gut Microbial Metabolite Contributing to Metabolic Syndrome in Children Based on Integrative Analyses of Microbiome-Metabolome Signatures. Microbiol. Spectr..

[20] Akhtar A.A., Turner D.P. (2022). The role of bacterial ATP-binding cassette (ABC) transporters in pathogenesis and virulence: Therapeutic and vaccine potential. Microb. Pathog..

[21] Vuong C., Otto M. (2002). *Staphylococcus epidermidis* infections. Microbes Infect..

[22] Foster T.J., Geoghe-gan J.A., Ganesh V.K., Höök M. (2014). Adhesion, invasion and evasion: The many functions of the surface proteins of *Staphylococcus aureus*. Nat. Rev. Microbiol..

[23] Patti J.M., Bremell T., Krajewska-Pietrasik D., Abdelnour A., Tarkowski A., Rydén C., Höök M. (1994). The *Staphylococcus aureus* collagen adhesin is a virulence determinant in experimental septic arthritis. Infect. Immun..

[24] Corrigan R.M., Rigby D., Handley P., Foster T.J. (2007). The role of *Staphylococcus aureus* surface protein SasG in adherence and biofilm formation. Microbiology.

[25] Rollof J., Braconier J.H., Söderström C., Nilsson-Ehle P. (1988). Interference of *Staphylococcus aureus* lipase with human granulocyte function. Eur. J. Clin. Microbiol. Infect. Dis..

[26] Sieprawska-Lupa M., Mydel P., Krawczyk K., Wójcik K., Puklo M., Lupa B., Suder P., Silber-ring J., Reed M., Pohl J. (2004). Degradation of human antimicrobial peptide LL-37 by *Staphylococcus aureus*-derived proteinases. Antimicrob. Agents Chemother..

[27] Sanz R., Marı N.I., Ruiz-Santa-Quiteria J.A., Orden J.A., Cid D., Diez R.M., Silhadi K.S., Amils R., de la Fuente R. (2000). Catalase deficiency in *Staphylococcus aureus* subsp. *anaerobius* is associated with natural loss-of-function mutations within the structural gene. Microbiology.

[28] Panmanee W., Hassett D.J. (2009). Differential roles of OxyR-controlled antioxidant enzymes alkyl hydroperoxide reductase (AhpCF) and catalase (KatB) in the protection of *Pseudomonas aeruginosa* against hydrogen peroxide in biofilm vs. planktonic culture. FEMS Microbiol. Lett..

[29] Liu B., Park S., Thompson C.D., Li X., Lee J.C. (2017). Antibodies to *Staphylococcus aureus* capsular polysaccharides 5 and 8 perform similarly in vitro but are functionally distinct in vivo. Virulence.

[30] Luo Z.Q., Isberg R.R. (2004). Multiple substrates of the *Legionella pneumophila* Dot/Icm system identified by interbacterial protein transfer. Proc. Natl. Acad. Sci. USA.

[31] De Pessemier B., López C.D., Taelman S., Verdonck M., Chen Y., Stockman A., Lambert J., Van de Wiele T., Callewaert C. (2025). Comparative Whole Metagenome Analysis in Lesional and Nonlesional Scalp Areas of Patients with Psoriasis Capitis and Healthy Individuals. J. Investig. Dermatol..

[32] De La Hoz-Romo M.C., Díaz L., Gómez-León J., Quintero M., Villamil L. (2025). Marine actinobacteria metabolites: Unlocking new treatments for acne vulgaris. Front. Microbiol..

[33] Harder J., Tsuruta D., Murakami M., Kurokawa I. (2013). What is the role of antimicrobial peptides (AMP) in acne vulgaris?. Exp. Dermatol..

[34] Li X., He C., Chen Z., Zhou C., Gan Y., Jia Y. (2017). A review of the role of sebum in the mechanism of acne pathogenesis. J. Cosmet. Dermatol..

[35] Callewaert C., Nakatsuji T., Knight R., Kosciolek T., Vrbanac A., Kotol P., Ardeleanu M., Hultsch T., Guttman-Yassky E., Bissonnette R. (2020). IL-4Rα Blockade by Dupilumab Decreases *Staphylococcus aureus* Colonization and Increases Microbial Diversity in Atopic Dermatitis. J. Investig. Dermatol..

[36] Vieira-Silva S., Sabino J., Valles-Colomer M., Falony G., Kathagen G., Caenepeel C., Cleynen I., van der Merwe S., Vermeire S., Raes J. (2019). Quantitative microbiome profiling disentangles inflammation- and bile duct obstruction-associated microbiota alterations across PSC/IBD diagnoses. Nat. Microbiol..

[37] Liang M.C., Li J.Q., Wu X.Y., Mo X.H., Ju Q. (2024). Analysis of hair follicle microbiota in non-lesional areas of patients with moderate-to-severe acne vulgaris: A single-center cross-sectional study. Shanghai Jiaotong Univ. Med. Sci..

[38] Guo Z., Yang Y., Wu Q., Liu M., Zhou L., Zhang L., Dong D. (2023). New insights into the characteristic skin microorganisms in different grades of acne and different acne sites. Front. Microbiol..

[39] Manurung T.H.P., Sitohang I.B.S., Agustin T. (2024). *Staphylococcus caprae* and *Staphylococcus epidermidis* define the skin microbiome among different grades of acne vulgaris. Arch. Dermatol. Res..

[40] Loss M., Thompson K.G., Agostinho-Hunt A., James G.A., Mongodin E.F., Rosenthal I., Cheng N., Leung S., Chien A.L., Kang S. (2021). Noninflammatory comedones have greater diversity in microbiome and are more prone to biofilm formation than inflammatory lesions of acne vulgaris. Int. J. Dermatol..

[41] Wang W., Dernst A., Martin B., Lorenzi L., Cadefau-Fabregat M., Phulphagar K., Wagener A., Budden C., Stair N., Wagner T. (2024). Butyrate and propionate are microbial danger signals that activate the NLRP3 inflammasome in human macrophages upon TLR stimulation. Cell Rep..

[42] Almoughrabie S., Cau L., Cavagnero K., O’Neill A.M., Li F., Roso-Mares A., Mainzer C., Closs B., Kolar M.J., Williams K.J. (2023). Commensal *Cutibacterium acnes* induce epidermal lipid synthesis important for skin barrier function. Sci. Adv..

[43] He Q., Shu H., Peng Y., Xu Y., Liu L., Zhou J., Zhao J., Xiong X., Li C. (2023). Untargeted metabolomics analysis of plasma metabolic characteristics in patients with acne and insulin resistance. Amino Acids.

[44] Belkaid Y., Hand T.W. (2014). Role of the microbiota in immunity and inflammation. Cell.

[45] O’Neill A.M., Gallo R.L. (2018). Host-microbiome interactions and recent progress into understanding the biology of acne vulgaris. Microbiome.

[46] Varin-Simon J., Colin M., Velard F., Tang-Fichaux M., Ohl X., Mongaret C., Gangloff S.C., Reffuveille F. (2024). *Cutibacterium acnes* biofilm formation is influenced by bone microenvironment, implant surfaces and bacterial internalization. BMC Microbiol..

[47] Dagnelie M.A., Corvec S., Timon-David E., Khammari A., Dréno B. (2022). *Cutibacterium acnes* and *Staphylococcus epidermidis*: The unmissable modulators of skin inflammatory response. Exp. Dermatol..

[48] Okoro O.E., Adenle A., Ludovici M., Truglio M., Marini F., Camera E. (2021). Lipidomics of facial sebum in the comparison between acne and non-acne adolescents with dark skin. Sci. Rep..

[49] Christensen G.J., Scholz C.F., Enghild J., Rohde H., Kilian M., Thürmer A., Brzuszkiewicz E., Lomholt H.B., Brüggemann H. (2016). Antagonism between *Staphylococcus epidermidis* and *Propionibacterium acnes* and its genomic basis. BMC Genom..

[50] Hamann T., Brüggemann H., Feidenhansl C., Rruci E., Gallinger J., Gallinat S., Hüpeden J. (2025). Distinct Intraspecies Variation of *Cutibacterium acnes* and *Staphylococcus epidermidis* in Acne Vulgaris and Healthy Skin. Microorganisms.

[51] Wang Y., Kuo S., Shu M., Yu J., Huang S., Dai A., Two A., Gallo R.L., Huang C.M. (2014). *Staphylococcus epidermidis* in the human skin microbiome mediates fermentation to inhibit the growth of *Propionibacterium acnes*: Implications of probiotics in acne vulgaris. Appl. Microbiol. Biotechnol..

[52] Ochlich D., Rademacher F., Drerup K.A., Gläser R., Harder J. (2023). The influence of the commensal skin bacterium *Staphylococcus epidermidis* on the epidermal barrier and inflammation: Implications for atopic dermatitis. Exp. Dermatol..

[53] Han H.S., Shin S.H., Choi B.Y., Koo N., Lim S., Son D., Chung M.J., Park K.Y., Sul W.J. (2022). A split face study on the effect of an anti-acne product containing fermentation products of *Enterococcus faecalis* CBT SL-5 on skin microbiome modification and acne improvement. J. Microbiol..

[54] Buchfink B., Xie C., Huson D.H. (2015). Fast and sensitive protein alignment using DIAMOND. Nat. Methods.

[55] Chen S., Zhou Y., Chen Y., Gu J. (2018). fastp: An ultra-fast all-in-one FASTQ preprocessor. Bioinformatics.

[56] Fu L., Niu B., Zhu Z., Wu S., Li W. (2012). CD-HIT: Accelerated for clustering the next-generation sequencing data. Bioinformatics.

[57] Hyatt D., Chen G.L., Locascio P.F., Land M.L., Larimer F.W., Hauser L.J. (2010). Prodigal: Prokaryotic gene recognition and translation initiation site identification. BMC Bioinform..

[58] Li D., Liu C.M., Luo R., Sadakane K., Lam T.W. (2015). MEGAHIT: An ultra-fast single-node solution for large and complex metagenomics assembly via succinct de Bruijn graph. Bioinformatics.

[59] Li H., Durbin R. (2009). Fast and accurate short read alignment with Burrows-Wheeler transform. Bioinformatics.

[60] Li R., Li Y., Kristiansen K., Wang J. (2008). SOAP: Short oligonucleotide alignment program. Bioinformatics.

[61] Noguchi H., Park J., Takagi T. (2006). MetaGene: Prokaryotic gene finding from environmental genome shotgun sequences. Nucleic Acids Res..

