# Supplementary material for: Integrated Metagenomic and Lipidomic Profiling Reveals Dysregulation of Facial Skin Microbiome in Moderate Acne Vulgaris
Source: Microorganisms. 2025 Nov 24;13(12):2674. doi: 10.3390/microorganisms13122674 (PMC12735717; doi:10.3390/microorganisms13122674)
Supplement: Supplementary file 1 [file microorganisms-13-02674-s001.zip › microorganisms-3941837-supplementary.pdf]

Supplement Figures S1-S8

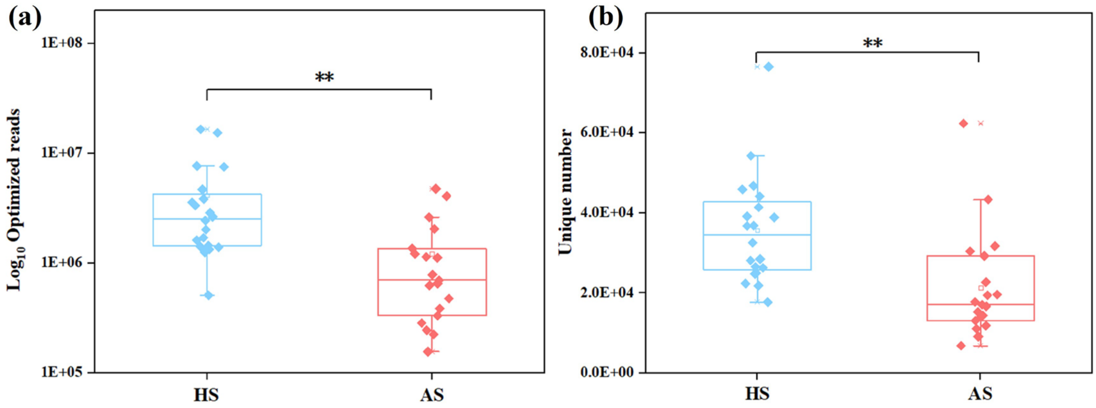

Figure S1. Comparison of sequencing abundance between acne group (AS) and healthy group (HS): (a) number of decontaminated reads after mapping trimmed reads against the non-redundant protein database (NR) using Burrows-Wheeler Aligner (BWA) v0.7.17, and (b) number of non-redundant genes aligning high-quality reads with the non-redundant gene set (default: 95% identity) using SOAPaligner v2.21. Asterisks indicate the significance level: $P < 0.01$ '**'.

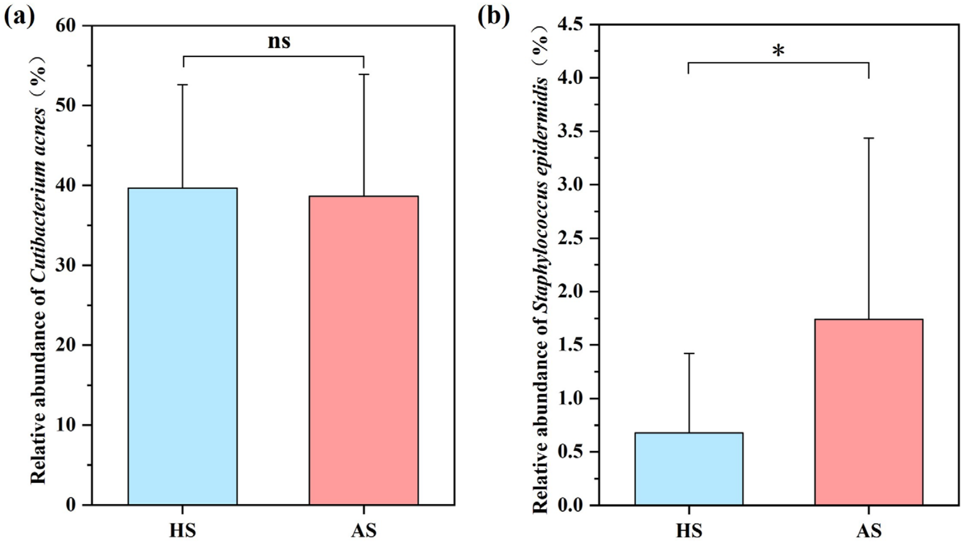

Figure S2. Relative abundances of *Cutibacterium acnes* and *Staphylococcus epidermidis* in AS and HS groups: (a) *C. acnes* and (b) *S. epidermidis*. Asterisks indicate significance levels: $P < 0.05$ '*'; ns for non-significant.

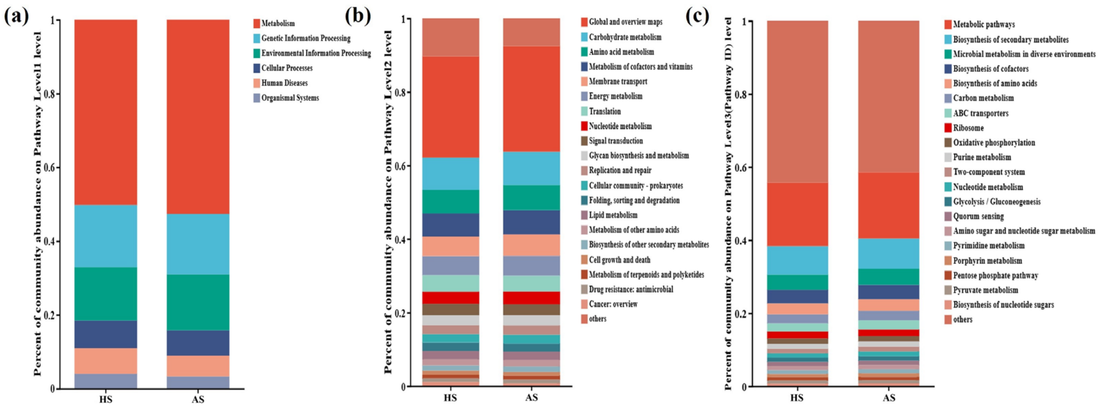

Figure S3. KEGG metabolic pathway composition in acne group (AS) and healthy group (HS): (a) Pathway level 1; (b) Pathway level 2; and (c) Pathway level 3.

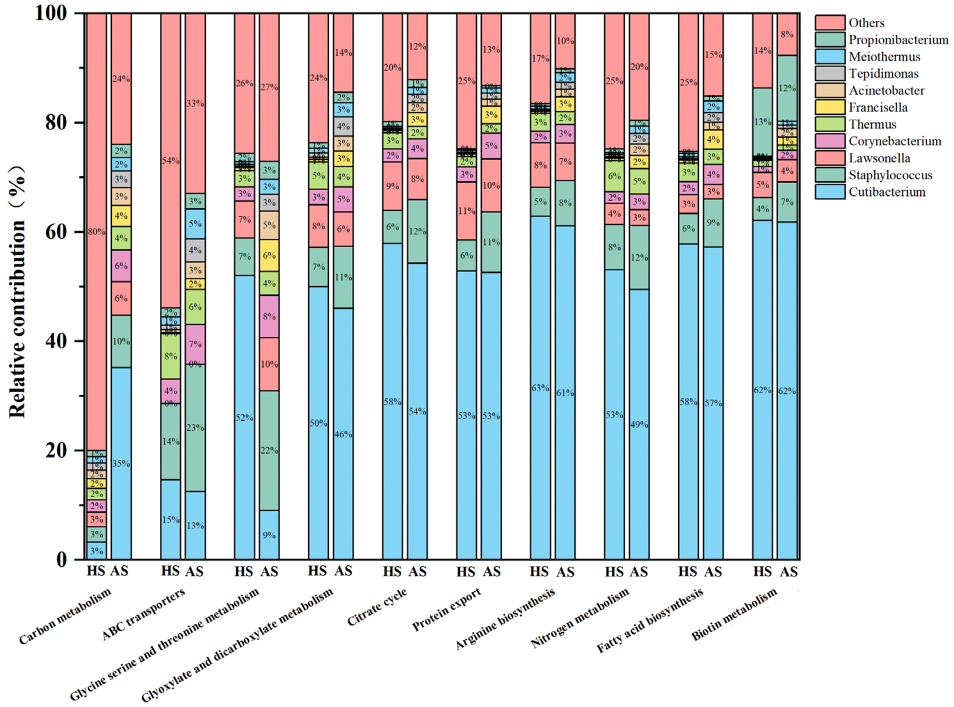

Figure S4. Contribution of bacterial genera to differences in KEGG pathways at level 3 between the acne group (AS) and healthy group (HS).

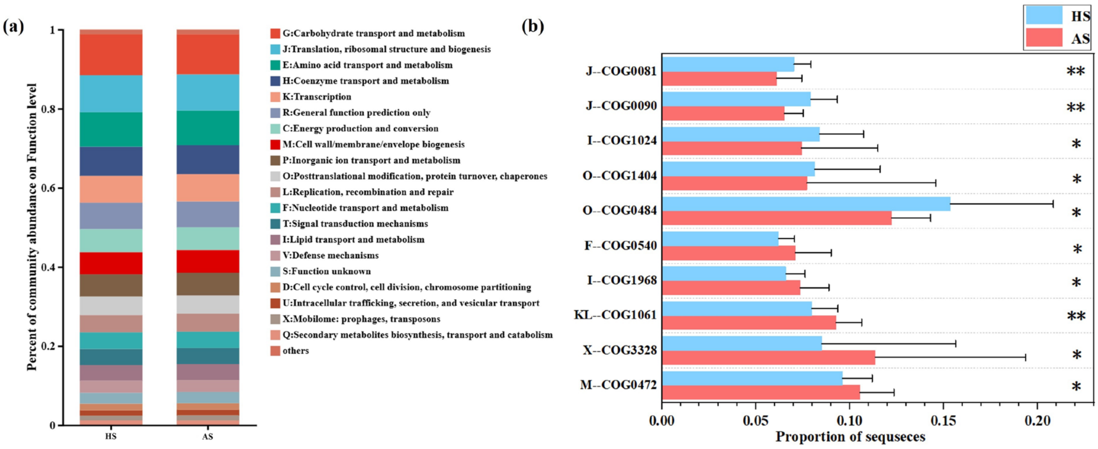

Figure S5. COG composition of facial microorganisms in acne group (AS) and health group (HS). (a) Relative abundance at the functional level in facial skin. (b) Significant difference in relative abundance at COG level. Asterisks indicate significance levels: P < 0.01 '**'; P < 0.05 '*'.

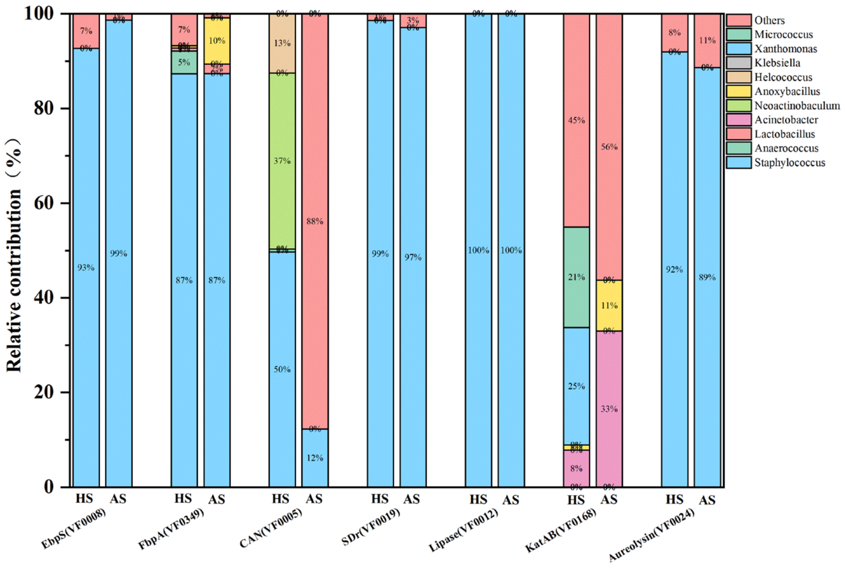

Figure S6. Contribution of bacterial genera to differences in virulence factors between the acne group (AS) and healthy group (HS).

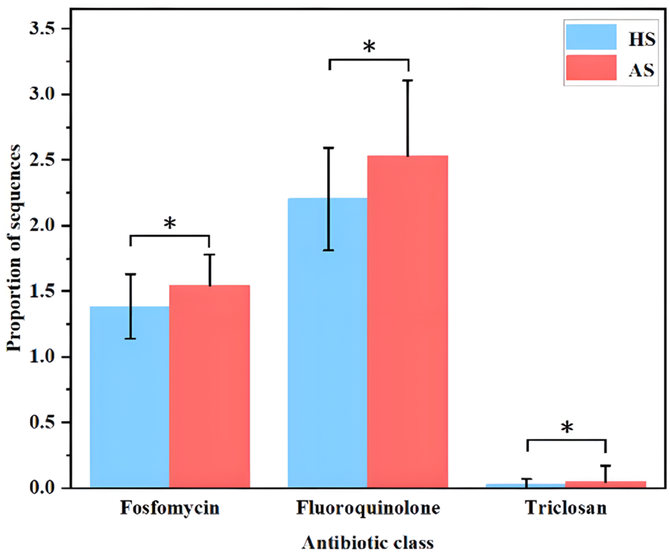

Figure S7. Differential antibiotic class composition between acne group (AS) and healthy group (HS). Asterisks indicate significance levels: P < 0.05 '*'.

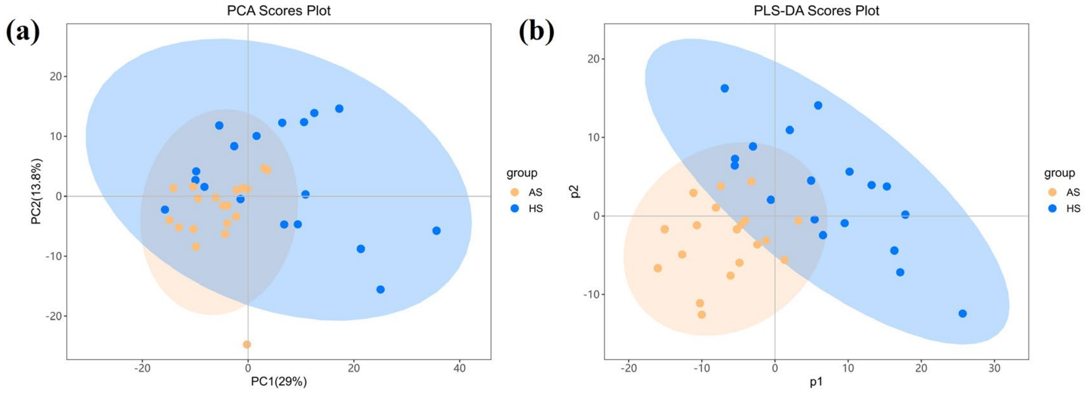

Figure S8. Metabolic profile differentiation between acne group (AS) and healthy group (HS). (a) Principal component analysis (PCA) scatterplot. (b) Partial least squares discriminant analysis (PLS-DA) scatterplot.

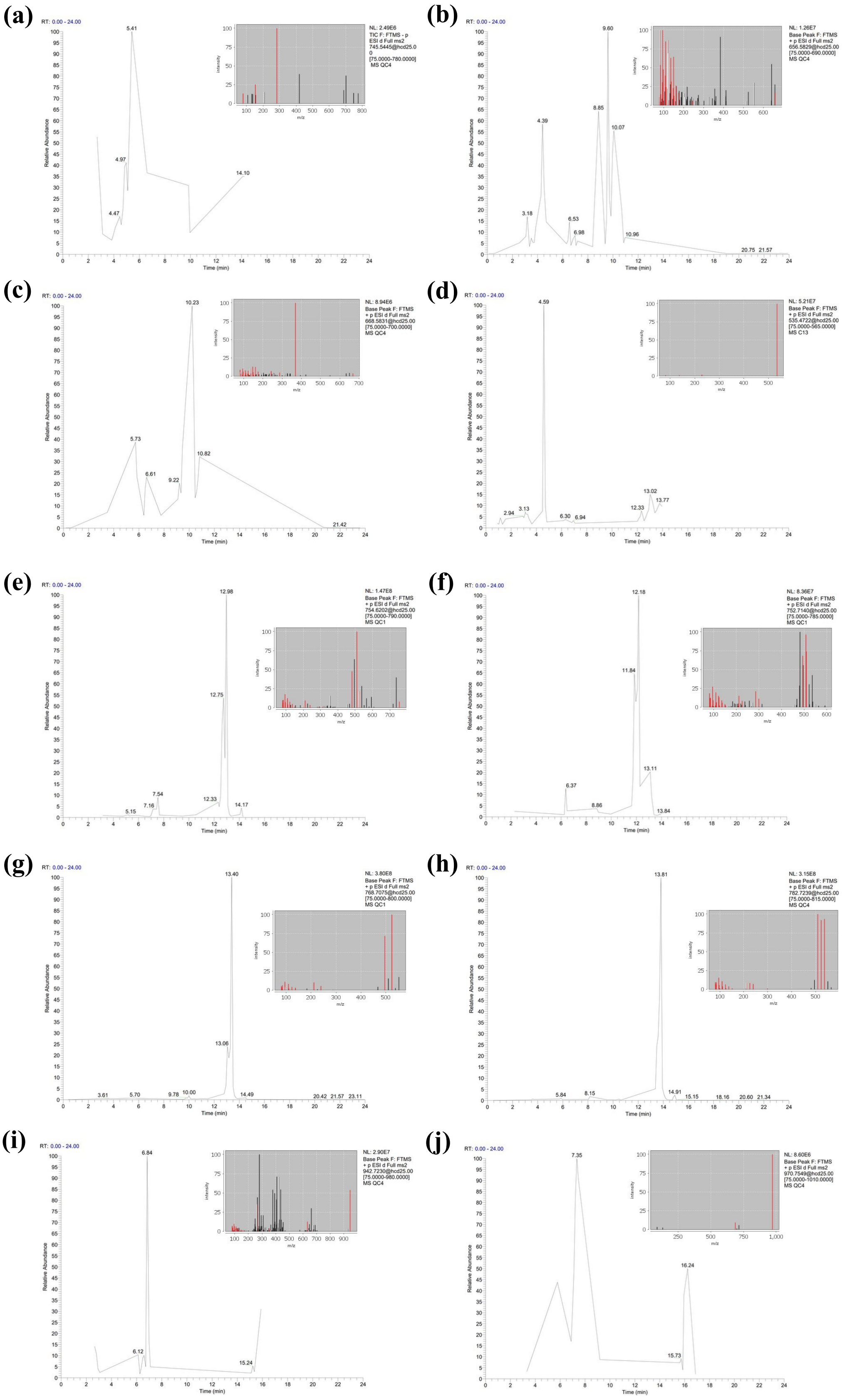

Figure S9. MS1 and MS2 Spectra of the Top 10 differential lipids between acne group (AS) and healthy group (HS). (a) PA(40:7)-H. (b) ChE(17:0)+NH4. (c) ChE(18:1)+NH4. (d) DG(30:3)+H. (e) TG(16:0_13:0_14:0)+NH4. (f) TG(15:0_14:0_14:1)+NH4. (g) TG(16:0_14:0_14:0)+NH4. (h) TG（15:0_14:0_16:0）. (i) TG(18:0e_20:2_20:2)+NH4. (g) TG(59:4)+NH4.

Supplementary Materials and Methods

1. Metagenomic sequencing

1.1. DNA extraction, library construction, and metagenomic sequencing

Total genomic DNA was extracted from skin swab samples using the QIAamp DNA Micro Kit (Qiagen, Hilden, Germany) according to the manufacturer's instructions. DNA concentration and purity were assessed with TBS-380 fluorometer and NanoDrop 2000 spectrophotometer, respectively. DNA quality was confirmed by 1% agarose gel electrophoresis.

DNA was fragmented to ~400 bp using Covaris M220 (Gene Company Limited, China) for paired-end library construction. Libraries were prepared using the NEXTFLEX Rapid DNA-Seq Kit (Bioo Scientific, Austin, TX, USA). Adapters containing full sequencing primer hybridization sites were ligated to blunt-ended fragments. Paired-end sequencing was performed on the Illumina NovaSeq platform (Illumina Inc., San Diego, CA, USA) at Majorbio Bio-Pharm Technology Co., Ltd. (Shanghai, China) using NovaSeq 6000 S4 Reagent Kit v1.5 (300 cycles), according to the manufacturer's instructions.

1.2. Sequence quality control and genome assembly

Raw reads were processed on the Majorbio Cloud Platform (www.majorbio.com). Adapter sequences were trimmed, and low-quality reads (length <50 bp, quality score <20, or containing N bases) were removed using fastp (Chen et al., 2018) (https://github.com/OpenGene/fastp, version 0.20.0). Reads mapping to the human genome were removed using Burrows-Wheeler Aligner (BWA) (Li et al., 2009) (http://bio-bwa.sourceforge.net, version 0.7.9a). Metagenomics assembly was performed using MEGAHIT (Li et al., 2015) (https://github.com/voutcn/megahit, version 1.1.2), which utilizes succinct de Bruijn graphs. Contigs ≥ 300 bp were retained for gene prediction and annotation.

1.3. Gene prediction, taxonomy, and functional annotation

Open reading frames (ORFs) were predicted from assembled contigs using Prodigal (Hyatt et al., 2010) and MetaGene (Noguchi et al., 2006) (http://metagene.cb.k.u-tokyo.ac.jp/). ORFs ≥ 100 bp were translated using the NCBI genetic code table (http://www.ncbi.nlm.nih.gov/Taxonomy/taxonomyhome.html/index.cgi?chapter=tgencodes#SG1). A non-redundant gene catalog was constructed using Cluster Database at High Identity with Tolerance (CD-HIT) (Fu et al., 2012) (http://www.bioinformatics.org/cd-hit/, version 4.6.1) at 90% identity and coverage thresholds. Reads were aligned to the catalog using SOAP aligner (Li et al., 2008) (http://soap.genomics.org.cn/, version 2.21) at ≥95% identity to quantify gene abundance. Representative sequences were taxonomically annotated by Diamond (Buchfink et al., 2015) (http://www.diamondsearch.org/index.php, version 0.8.35) alignment against the NCBI NR database (e-value $\leq 1e^{-5}$). Functional annotations were performed against eggNOG (COG) and Kyoto Encyclopedia of Genes and Genomes (KEGG) databases with the same e-value cutoff. Antibiotic resistance and virulence factor genes were identified via Diamond alignment against CARD and VFDB databases

(https://card.mcmaster.ca/home), respectively, using the same threshold.

2. Untargeted lipidomic analysis

2.1. Lipid extraction

After thawing at 4 °C for 20 min, the Sebutape® patch sample was placed in a 1.5 mL microcentrifuge tube. Then, 200 μL ultrapure water and 240 μL precooled methanol were added sequentially, with gentle mixing after each addition using an IKA vortex at 500 rpm (IKA-Werke GmbH & Co. KG, Staufen, Germany). Subsequently, 800 μL of methyl tert-butyl ether (MTBE) was added, and the mixture was ultrasonicated in an ice-cold water bath at 4 °C for 20 min. After equilibrating at 25 °C for 30 min, the samples were centrifuged at 14,000 × g for 15 min at 10 °C. Approximately 500 μL of the upper organic phase was collected, dried under nitrogen using a Termovap concentrator, and stored at –80 °C until Liquid Chromatography-Tandem Mass Spectrometry (LC-MS/MS) analysis.

2.2. Liquid Chromatography-Tandem Mass Spectrometry (LC-MS/MS) analysis

Lipid separation was performed by reversed-phase liquid chromatography using a Waters CSH C18 column (1.7 μm, 2.1 mm × 100 mm). Lipid extracts were reconstituted in 200 μL of 90% isopropanol/acetonitrile, centrifuged at 14000 × g for 15 min, and 3 μL of the supernatant was injected. Solvent A consisted of acetonitrile-water (6:4, v/v) with 0.1% formic acid and 0.1 mM ammonium formate, and solvent B was acetonitrile-isopropanol (1:9, v/v) with the same additives. The initial mobile phase consisted of 40% solvent B at a flow rate of 300 μL/min, held for 3.5 min, then increased linearly to 75% over 9.5 min and to 99% over 6 min, followed by equilibration at 40% solvent B for 5 min.

Mass spectra were acquired on a Q-Exactive Plus in positive and negative electrospray ionization modes. Source parameters were: source temperature, 300 °C; capillary temperature, 350 °C; spray voltage, 3000 V; S-Lens RF level, 50%; and scan range, m/z 200–1800.

2.3. Lipid identification

LipidSearch software (Thermo Scientific™) was used for lipid identification, peak extraction, comparison, and quantification. Its database contains ~1.7 million lipid species across 300 subclasses within eight lipid classes. High-resolution Q-Exactive Plus data enabled accurate identification based on precursor, product, and neutral loss scans. Mass tolerance for precursor, product, and fragment ions was set at 5 ppm to enhance selectivity and minimize false positives.

References

1. Buchfink B, Xie C, Huson DH. Fast and sensitive protein alignment using DIAMOND. Nat Methods 2015;12(1):59-60.
2. Chen S, Zhou Y, Chen Y, Gu J. fastp: an ultra-fast all-in-one FASTQ preprocessor. Bioinformatics 2018;34(17): i884-i890.
3. Fu L, Niu B, Zhu Z, Wu S, Li W. CD-HIT: accelerated for clustering the next-generation sequencing data. Bioinformatics 2012;28(23):3150-2.
4. Hyatt D, Chen GL, Locascio PF, Land ML, Larimer FW, Hauser LJ. Prodigal: prokaryotic gene recognition and translation initiation site identification. BMC Bioinformatics 2010; 11:119.
5. Li D, Liu CM, Luo R, Sadakane K, Lam TW. MEGAHIT: an ultra-fast single-node solution for large and complex metagenomics assembly via succinct de Bruijn graph. Bioinformatics 2015;31(10):1674-6.
6. Li H, Durbin R. Fast and accurate short read alignment with Burrows-Wheeler transform. Bioinformatics 2009;25(14):1754-60.
7. Li R, Li Y, Kristiansen K, Wang J. SOAP: short oligonucleotide alignment program. Bioinformatics 2008;24(5):713-4.
8. Noguchi H, Park J, Takagi T. MetaGene: prokaryotic gene finding from environmental genome shotgun sequences. Nucleic Acids Res 2006;34(19):5623-30.
